# Supplementary material for: Selenium Species and Fractions in the Rock–Soil–Plant Interface of Maize (Zea mays L.) Grown in a Natural Ultra-Rich Se Environment
Source: Int J Environ Res Public Health. 2023 Feb 24;20(5):4032. doi: 10.3390/ijerph20054032 (PMC10001709; doi:10.3390/ijerph20054032)
Supplement: Supplementary file 1 [file ijerph-20-04032-s001.zip › ijerph-2178892-supplementary.pdf]

Selenium Species and Fractions in the Rock–Soil–Plant Interface of Maize (*Zea mays* L.) Grown in a Natural Ultra-Rich Se Environment

Diego Armando Pinzon-Nuñez [1,2,3], Oliver Wiche [4], Zhengyu Bao [1,5,6], Shuyun Xie [2], Bolun Fan [1,7], Wenkai Zhang [1], Molan Tang [1,8,*] and Huan Tian [1,3,*]

1 Faculty of Materials Science and Chemistry, China University of Geosciences, Wuhan 430074, China
2 School of Earth Sciences, China University of Geosciences, Wuhan 430074, China
3 Ziyang Zhongdida Selenium Technology Co., Ltd., Ankang 72500, China
4 Biology/Ecology Unit, Institute of Biosciences, Technische Universität Bergakademie Freiberg, 09599 Freiberg, Germany
5 Zhejiang Institute, China University of Geosciences, Hangzhou 311305, China
6 Ankang Se-Resources Hi-Tech Co., Ltd., Ankang 725000, China
7 Scientific Research Academy of Guangxi Environment Protection, Nanning 530022, China
8 New Generation Information Technology Research Institute, Guangxi Academy of Sciences, Nanning 530007, China
* Correspondence: molan_tang@cug.edu.cn (M.T.); huantian@cug.edu.cn (H.T.)

Table of Contents

1. **HPLC-ICP-MS method optimization**........ 2

1.1. Citric acid concentration
1.2. Methanol concentration
1.3. pH of citric acid
1.4. Standard curve and detection limit
1.5. Selection of sample enzymolysis method
1.6. Evaluation of the extraction efficiency of selenium species
1.7. Method test

2. **Supplementary material results**........ 6

2.1. **Table S2a**. Test result of SeMet in ERM-BC201a (*n* = 3)
2.2. **Table S2b.** Experimental results of spiked recovery of five Se species in ERM-BC201a
2.3. **Table S3.** The Se fractions results of soils and rock parental material analyzed.
2.4. **Table S4.** Pearson correlations between Se fractions and tSe.
2.5. **Table S5.** Spearman correlations between Se fractions and tSe.
2.6. **Table S6.** Pearson correlations between tSe and biomass in maiz organs, and tSe and Se soil fractions.
2.7. **Table S7a**. Selenium species concentrations (mg kg^{-1}) in maize's grain and leaf.
2.8. **Table S7b.** Selenium species concentrations (mg kg^{-1}) in maize's stalk and root.

Supplementary material:

1. HPCL-ICP-MS method optimization

The stationary phase was the anion-exchange column, while the mobile phase was ammonium citrate and methanol. To modify concentration, ultra-pure water, methanol, and 50 mmol L^{-1} citric acid were mixed in a specified proportion. Se(IV), Se(VI), SeMet, SeCys2, and MeSeCys were diluted at 10 μg L^{-1}. The concentration of citric acid, methanol and pH value of citric acid in HPLC mobile phase were optimized as follows:

1.1. Citric acid concentration

Percentages of 5%, 10%, 15%, and 20% citric acid (pH 5.5, 50 mmol/L) were optimized (Figure S1). As citric acid concentration increases, Se(IV) and Se(VI) peak earlier and overlap more with organic selenium form, making them harder to differentiate. Therefore, 5% (2.5 mmol L^{-1}) or 10% (5 mmol L^{-1}) citric acid was more satisfactory, and the separation degree among peaks was the maximum, and the peak time of 10% citric acid was within 10 min. Therefore, we considered 10% (5 mmol L^{-1}) citric acid ratio (concentration).

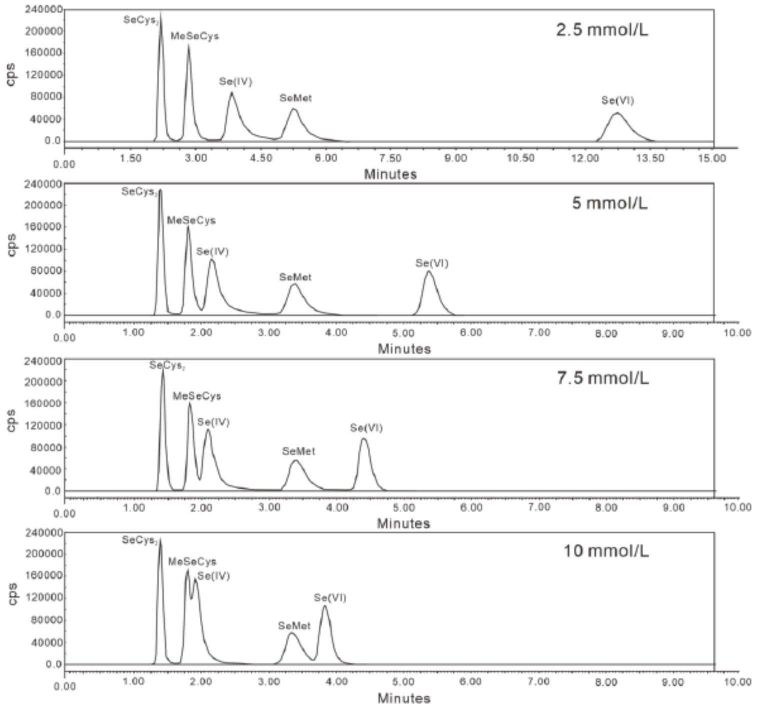

Figure S1. Chromatogram of 10 μg L^{-1} Se at different citric acid concentrations.

1.2. Methanol concentration

Percentages of 1%, 2%, 3% methanol and 10% citric acid (pH 5.5, 50 mmol L^{-1}) were optimized (Figure S2). Methanol concentrations of 2% and 3% produced the best peak effect and peak separation.

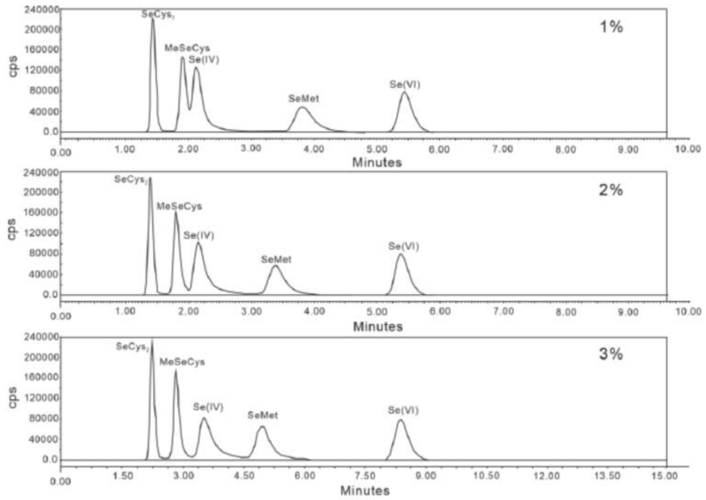

Figure S2. Chromatograms of 10 μg L^{-1} Se at different methanol percentages.

1.3. pH of citric acid

For optimization of pH , 87% ultra pure water, 3% methanol, and 10% citric acid (50 mmol L^{-1}) were chosen (Figure S3). The citric acid peak output impact at pH 5 and 5.5 is optimal. Peak separation was largest, while citric acid with pH = 5.5 peaked in less than 10 min. Hence, pH 5.5 citric acid was chosen. In summary, HPLC mobile phases were a mixture of 88% ultrapure water, 3% methanol and 10% citric acid 50 mmol L^{-1} with pH = 5.5.

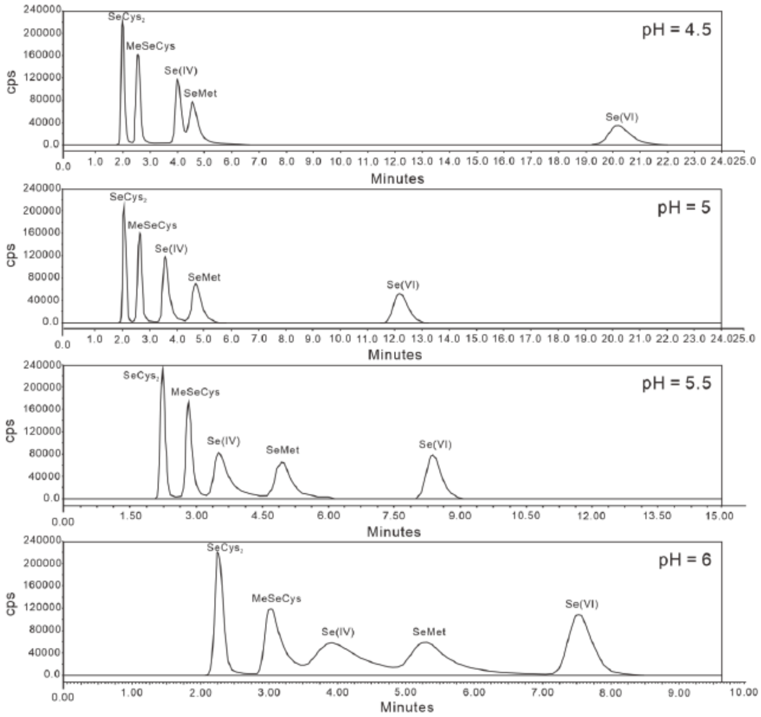

Figure S3. Chromatograms of 10 g L^{-1} Se species at different pH of citric acid

1.4. Standard curve and detection limit

Under the method adopted in this study, the mixed standard solutions of five kinds of selenium with gradient of 1, 2.5, 5, 10, 25, 50 μg L^{-1} were determined. The method's detection limit was estimated using Formula 1 after adding a specified amount of standard solution (anticipated to be 2-5 times the detection limit) to the sample.

$$DL = t * S \quad (1)$$

Where, t is the 99% confidence of the research value and the standard deviation of the estimation using n-1 degree of freedom. When the measurement is repeated for 7 times, t = 3.143. The limits of detection of the five selenium forms found by this approach ranged from 0.87 to 1.70 μg L^{-1}, with SeCys2 being the lowest and SeMet the highest. The detection limit obtained by this method is similar to that obtained by other HPLC-ICP-MS methods.

1.5. Selection of sample enzymolysis method

Since the extraction step is crucial for accurate species determination, the extraction efficiency was evaluated by a comparison of three extraction protocols avialable in the literature, the ERM-BC210a standard material matching method, the Selenium-enriched Agricultural Products standards method of China Supply and Marketing Cooperation General Association (GH/T 1135-2017) [1], and the third method was a slight modification of the latter, using only protease XIV and following the digestion process in Bañuelos et al. [2]. Specific methods and steps were as follows:

Method I. According to the European Union ERM-BC210a standard material, 0.5 g sample was weighed in triplicates into 20 mL centrifuge tubes, and 60 mg protease XIV, 30 mg lipase and 10 mL 30 Mm Tris-HCl (pH = 7.5) were added for ultrasonic degassing. After that, they were cultured in darkness at 37°C for 20 hours, mixed well, and oscillated in a water bath oscillator at 150 r/min. The hydrolyzed sample was centrifuged at 10000 r/min at 4°C for 30 min. The supernatant was filtered and stored at -20 °C. The remaining residue was added to 5 mL of hydrolysate containing 100 mg of driselase, and the above steps were repeated. The two supernatants were combined and diluted and immediately injected in a HCLP-ICP-MS.

Method II. A portion of 0.5g of sample were placed into 20 mL centrifuge tubes, added 5 mL Tris-HCl, and after shaked placed in ultrasonic during 30 min, then 50 mg cellulase were added, and 20 mg of protease K, after shaking the solution was placed in water bath at constant of 50°C, and rotation speed of 250 r/min. After culturing for 18 h, 20 mg protease XIV was added and centrifuged for 30 min at 10000 r/min at 4°C for 18 h. The supernatant was filtered through 0.22 μm membrane and immediately injected in the HPLC-ICP-MS.

Method III. A portion of 0.5 g sample were placed into 20 mL centrifuge tube, add 5 mL TrIS-HCl was added and placed in ultrasound for 30 min, in this step 25 mg protease XIV was added, and kept in a costant culturing temparature in darkness of 37°C, and shaked at 150

r/min, for 24 h, then centrifuged at 4°C for 30 min at 10000 r/min. The supernatants were filtered through 0.22 μm membrane and immediately analyzed by HPLC-ICP-MS.

The test results of the three methods are shown in Table **S1**. In terms of enzymolysis rate, the average range of enzymolysis rate of samples extracted by method I was 90.96%, that extracted by method II was 85.76% and that extracted by method III was 63.90%. The extraction efficiency of the first and second methods were ideal. MeSeCys and Se(IV) test results showed that the detection limit was near, the peak was unstable and the RSD was large, which will not be discussed here. The three enzyme extraction methods had good repeatability for SeMet and Se(VI) in the samples with high selenium content

Table S1. Results of the different methods for testing the reference material.

Method	SeCys2	MeSeCys	Se(IV)	SeMet	Se(VI)	∑Species	tSe	Recovery (%)
I	0.76 ± 0.03a	0.43 ± 0.17a	0.25 ± 0.13a	10.2 ± 0.1a	0.54 ± 0.02a	16.0 ± 1.2a	18.7 ± 0.4ab	85.8 ± 4.5a
II	0.72 ± 0.16a	0.25 ± 0.06a	0.28 ± 0.07a	9.0 ± 0.2b*	0.48 ±0.01b	18.6 ±1.2a	19.5 ± 1.4a	90.9 ± 9.1a
III	0.74 ± 0.01a	0.15 ± 0.03b	0.17 ± 0.06a	6.7 ± 0.1c**	0.37 ± 0.01c	11.2 ± 0.5b	18.6 ± 1.3b	63.9 ± 4.6b

1.6. Evaluation of the extraction efficiency of Selenium species

Method I (85.8%) and Method II (90.9%) had similar enzymolysis recoveries. Method III had much lower recoveries (63.9%) ($p < 0.01$) than Methods I and II, likely due to incomplete hydrolysis and/or considerable element losses of one or more Se species. The observed amounts of SeCys, Se(IV), and SeMet were comparable across all extraction procedures, however Method III yielded 40% less MeSeCys and 26% less SeMet than Method II and 65% less and 34% less in the extraction solutions. Methods I and II yielded SeMet concentrations that were not substantially different from the certified values (11.03 ± 1.05 μg g^{-1}), whereas Method III yielded a significant difference at $\alpha = 0.1\%$.

Method I measured SeCys2, SeMet, and Se(VI) greater than Method II. Method II's proteinase K needs 50-70°C to be active, and SeMet's thermal stability is weak at high temperatures, therefore the long-term high temperature during enzymatic hydrolysis causes SeMet's morphological transformation [3]. Shi et al. [4] recently also found that combining proteinase XIV and protease K did not change significantly the extraction. Then for practicity and to follow the standard method of the reference material, this paper used Method I to extract enzymatically hydrolyze plant Se.

1.7. Method test

Figure S4 shows the selected method's ERM-BC210a standard form chromatogram. The standard substance certificate's SeMet mass fraction is 27.4 ± 2.6 mg kg^{-1}, while Se's is 11.03 ± 1.05 mg kg^{-1}. The average SeMet content was 10.21 mg kg^{-1}, which was not significantly different from the standard value of 11.03 mg kg^{-1} ($P < 0.05$), and the RSD of the test result was 0.73%, indicating that the method is precise and accurate and can extract and detect the

selenium form in selenium-rich plants (Table S1). Table S2 shows a conventional recovery experiment for five selenium forms in ERM-BC210a sample to test the method's reliability.

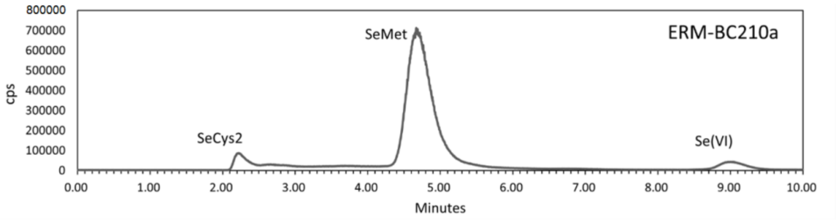

Figure S4. Chromatograms of Se species in ERM-BC210a standard.

2. Supplementary material of results mentioned in the paper:

2.1. **Table S2a**. Test result of SeMet in ERM-BC201a (n = 3)

Se form	Unit	ERM-BC201a	
		Standard value ± uncertainty	Determined value ± SD
SeMet	mg/kg	11.03 ± 1.05	10.21 ± 0.08

2.2. **Table S2b.** Experimental results of spiked recovery of five Se species in ERM-BC201a

Se form	Background (mg/kg)	Spike amount (mg/kg)	Measured Value (mg/kg)	Recovery (%)
	0.76	0.50	1.25	98.0
SeCys2	0.79	1.00	1.83	104.0
	0.74	2.00	2.56	91.0
	0.25	0.50	0.74	98.0
MeSeCys	0.59	1.00	1.64	105.0
	0.46	2.00	2.39	96.5
	0.16	0.50	0.63	94.0
Se(IV)	0.19	1.00	1.12	93.0
	0.41	2.00	2.32	95.5
	10.30	0.50	10.73	86.0
SeMet	10.17	1.00	11.25	108.0
	10.17	2.00	12.06	94.5
	0.53	0.50	0.98	90.0
Se(VI)	0.56	1.00	1.51	95.0
	0.54	2.00	2.47	96.5

pH	TOC	Type/Site	F1	F2	F3	F4	F5	Sum	tSe	Recovery	SD
7.54	4.47	Soil1	0.09	0.16	0.40	4.04	10.03	14.72	13.30	110.63	0.61
7.71	3.31	Soil2	0.05	0.14	0.25	2.12	6.51	9.07	8.58	105.75	0.34
7.9	4.93	Soil3	0.08	0.09	0.57	7.66	19.58	27.99	29.38	95.26	2.98
7.03	5.72	Soil4	0.13	0.46	0.67	4.23	17.50	22.99	21.99	104.53	4.12
7.7	7.03	Soil5	0.09	0.14	0.99	4.77	21.15	27.14	29.95	90.61	2.20
7.7	2.24	Soil6	0.07	0.21	0.57	1.46	4.75	7.06	7.81	90.47	0.11
7.69	5.05	Soil7	0.05	0.28	0.79	1.32	4.87	7.30	8.00	91.32	0.62
7.35	3.21	Soil8	0.07	0.25	0.81	1.48	4.46	7.07	8.83	80.09	0.73
6.47	5.24	Soil 9	0.08	0.26	0.76	2.24	6.67	10.01	11.12	90.07	0.76
7.59	4.28	Soil10	0.09	0.17	0.50	7.65	17.50	25.92	27.80	93.22	1.22
7.6	5.19	Soil11	0.05	0.15	0.37	1.36	13.41	15.34	14.23	107.83	1.77
9.33	1.93	Rock12	0.02	0.22	0.16	1.43	0.19	2.03	2.02	100.76	0.24
9.04	2.05	Rock13	0.05	0.39	1.06	7.41	0.51	9.41	9.86	95.49	0.44
8.5	1.07	Rock14	0.16	0.69	4.83	38.60	0.59	44.86	41.46	108.21	0.56
8.73	8.41	Rock15	0.03	0.08	0.57	7.84	0.38	8.91	7.73	115.24	6.77
8.04	13.12	Rock16	7.81	11.24	22.86	28.69	19.99	90.58	85.48	105.97	4.13
8.32	3.42	Rock17	0.00	0.08	3.18	0.09	0.58	3.92	3.85	98.21	0.48
9.14	3.06	Rock18	0.13	0.50	0.58	5.53	0.72	7.46	6.09	0.13	0.26
9.35	8.13	Rock19	0.02	0.28	0.30	0.71	0.19	1.50	1.30	0.02	0.57
9.32	3.82	Rock20	0.00	0.47	0.13	3.22	0.44	4.26	3.69	115.38	1.05
6.53	14.97	Rock21	0.25	2.29	21.68	19.51	4.4	48.13	40.87	117.78	1.10
8.8	0.22	Rock 22	0.06	0.20	0.71	7.11	0.89	8.97	7.74	0.06	4.00

2.3. **Table S3.** The Se fractions results of soils and rock parental material analyzed.

Note : F1: water soluble Se, F2: Exchangable Se, F3: Alkali-soluble Se, F4: Acid soluble Se, and F5: Residual Se. Fractions are given in Se mg kg^{-1}, while TOC and recoveries are given in percentages.

2.4. **Table S4.** Pearson correlations between Se fractions and tSe.

Pearson	SF2	S-tSe	R-tSe	SF1+F2	SF4+F5	RF1-F2	RF3-F4	RF4-F5	tSe-Root	tSe-Stalk	tSe-Leaf	tSe-Grain
S-F2	1	-0.211	0.019	0.983**	-0.206	-0.275	-0.458	-0.451	0.337	0.568	0.701*	0.524
S-tSe		1	0.275	-0.058	0.986**	0.556	0.906**	0.828**	0.177	-0.079	-0.127	-0.201
R-tSe			1	0.031	0.339	0.254	0.123	0.195	-0.491	-0.304	-0.397	-0.581
SF1+F2				1	-0.051	-0.197	-0.340	-0.341	0.380	0.579	0.691*	0.541
SF4+F5					1	0.481	0.852**	0.829**	0.156	-0.106	-0.178	-0.273
RF1+F2						1	0.685*	0.754*	0.022	-0.237	-0.153	-0.045
RF3+F4							1	0.969**	0.150	-0.213	-0.196	-0.217
RF4+F5								1	0.140	-0.220	-0.212	-0.230
tSe-Root									1	0.685*	0.76*	0.653*
tSe-Stalk										1	0.701*	0.777*
tSe-Leaf											1	0.684*
tSe-Grain												1

Note: For both tables (*$p < 0.05$, **$p < 0.01$, correlation coefficient), S and R mean soil and rock, respectively.

2.5. **Table S5.** Spearman correlations between Se fractions and tSe.

Spearman	SF2	S-tSe	R-tSe	SF1+F2	SF4+F5	RF1-F2	RF3-F4	RF4-F5	tSe-Root	tSe-Stalk	tSe-Leaf	tSe-Grain
S-F2	1	-0.464	-0.327	0.964**	-0.536	-0.400	-0.564	-0.473	0.527	0.682*	0.781*	0.709*
S-tSe		1	0.709*	-0.327	0.927**	0.518	0.664	0.855**	-0.118	-0.191	-0.273	-0.464
R-tSe			1	-0.255	0.791**	0.073	0.582	0.655*	-0.091	-0.200	-0.191	-0.382
SF1+F2				1	-0.427	-0.300	-0.509	-0.400	0.555	0.691*	0.791	0.800
SF4+F5					1	0.436	0.773*	0.864**	0.018	-0.073	-0.200	-0.491
RF1+F2						1	0.609*	0.664*	0.091	-0.055	-0.136	-0.255
RF3+F4							1	0.918**	0.000	-0.136	-0.255	-0.391
RF4+F5								1	0.000	-0.127	-0.236	-0.464
tSe-Root									1	0.909**	0.909**	0.482
tSe-Stalk										1	0.918**	0.582
tSe-Leaf											1	0.609*
tSe-Grain												1

Note: For both tables (*$p < 0.05$, **$p < 0.01$, correlation coefficient), S and R mean soil and rock, respectively.

2.6. **Table S6.** Pearson correlations between tSe and biomass in maiz organs, and tSe and Se soil fractions.

Pearson	tSe-Root	Root-B	tSe-Stalk	Stalk-B	tSe-Leaf	Leaf-B	tSe-Grain	Grain-B	tSe-soil	SF1	SF2	SF3	SF4	SF5
tSe-Root	1	-0.412	0.678**	-0.060	0.756**	-0.320	0.629**	-0.300	0.177	0.257	0.320	0.239	0.111	0.018
Root-B		1	-0.320	-0.136	-0.533*	0.361*	-0.220	0.501*	-0.371*	-0.403*	-0.344*	-0.697**	-0.231	-0.332
tSe-Stalk			1	0.089**	0.688**	0.043	0.767**	-0.346*	-0.077	0.293	0.547**	0.296	-0.112	-0.181
Stalk-B				1	0.106	-0.112	-0.295	0.372*	0.389*	0.127	0.130	0.072	0.381*	0.280
tSe-Leaf					1	-0.036	0.659**	-0.198	-0.133	0.214	0.686**	0.502*	-0.214	-0.252
Leaf-B						1	0.053	-0.085	-0.672**	-0.663**	-0.182	0.049	-0.590**	-0.606**
tSe-Grain							1	-0.441*	-0.195	0.243	0.4816*	0.382*	-0.284	-0.348*
Grain-B								1	-0.227	-0.217	-0.020	-0.626**	-0.008	-0.274
tSe-soil									1	0.559	-0.168	0.258	0.757**	0.862**
SF1										1	0.487*	0.246	0.542*	0.611**
SF2											1	0.329*	-0.302*	-0.173
SF3												1	-0.072	0.098
SF4													1	0.828**
SF5														1

Note: S represents soils and B represents biomasses (*$p < 0.05$, **$p < 0.01$, correlation coefficient).

2.7. **Table S7a**. Selenium species concentrations (mg kg^{-1}) in maize's grain and leaf.

Organ	SeCys2	MeSeCys	SeMet	Se(IV)	Se(VI)	Sum	tSe	Recovery
Grain 1	0.24	0.19	4.25	0.02	0.04	4.74	4.81	98.54
Grain 2	0.52	0.08	5.04	0.03	0.02	5.7	5.33	106.90
Grain 3	0.3	0.12	4.41	0.02	0.01	4.86	4.5	108.07
Grain 4	0.47	0.09	6.20	0.04	0.07	6.87	7.35	93.45
Grain 5	0.29	0.13	5.47	0.02	0.01	5.93	5.47	108.45
Grain 6	0.57	0.2	7.2	0.03	0.01	8.02	7.88	101.71
Grain 7	0.52	0.12	5.01	0.06	0.03	5.75	5.51	104.30
Grain 8	0.44	0.13	4.79	0.02	0.02	5.39	5.9	91.47
Grain9	0.77	0.28	8.91	0.03	0.03	10.01	9.41	106.38
Grain 10	0.40	0.08	3.41	0.04	0.04	3.96	5.05	78.51
Grain 11	0.19	0.03	0.62	0.01	< dl	0.85	0.97	87.81
Leaf 1	0.15	0.19	2.95	< dl	0.07	3.36	12.41	27.08
Leaf 2	0.28	0.56	2.85	0.04	0.06	3.8	4.73	80.33
Leaf 3	0.31	1.8	5.44	0.03	0.16	7.74	12.36	62.62
Leaf 4	0.44	2.5	9.49	0.04	0.35	12.81	24.56	52.18
Leaf 5	0.29	0.92	6.61	0.07	0.35	8.23	11.03	74.65
Leaf 6	0.49	1.8	7.19	0.11	0.25	9.83	19.03	51.65
Leaf 7	0.58	2.4	11.96	0.15	0.79	15.88	29.56	53.72
Leaf 8	0.26	0.91	5.1	0.03	0.14	6.44	11.92	53.99
Leaf 9	0.45	1.08	10.5	0.13	0.48	12.64	22.42	56.4
Leaf 10	0.34	1.27	5.46	0.03	0.03	7.13	14.36	49.64
Leaf 11	0.04	0.1	0.27	< dl	0.02	0.43	1.32	32.38

Note: For both speciation results tables. <dl indicates that the content is lower than the detection limit. Recovery is given in percentages. Table entries for organic species show selenium as selenium amino acid content, hence percentages may differ from those shown for inorganic species. The following formula can be used to convert to Se-content: Se-SeMet= SeMet*0.4026, Se-SeCys2= SeCys2*0.4727, Se-MeSeCys= MeSeCys*0.4699.

2.8. **Table S7b.** Selenium species concentrations (mg kg^{-1}) in maize's stalk and root.

Organ	SeCys2	MeSeCys	SeMet	Se(IV)	Se(VI)	Sum	tSe	Recovery
Stalk 1	0.19	0.1	0.47	< dl	0.11	0.87	2.71	31.96
Stalk 2	0.14	0.25	0.71	< dl	0.09	1.19	2.43	48.9
Stalk 3	0.1	0.21	0.82	< dl	0.28	1.42	3.33	42.60
Stalk 4	0.29	0.75	1.18	< dl	0.39	2.61	5.48	47.53
Stalk 5	0.1	0.05	0.37	0.01	0.02	0.55	2.16	25.63
Stalk 6	0.13	0.27	0.84	< dl	0.08	1.32	3.36	39.28
Stalk 7	0.13	0.43	0.86	< dl	0.21	1.62	3.99	40.53
Stalk 8	0.14	0.14	0.45	< dl	0.05	0.79	2.27	34.53
Stalk 9	0.42	0.98	2.30	< dl	0.72	4.41	8.26	53.39
Stalk 10	0.13	0.05	0.78	< dl	0.06	1.03	3.42	30.15
Stalk 11	0.06	0.04	0.13	< dl	0.02	0.26	1.38	18.50
Root 1	0.03	0.05	0.17	0.02	< dl	0.27	7.74	3.52
Root 2	0.07	0.47	0.5	0.03	< dl	1.07	6.95	15.37
Root 3	0.12	0.37	0.48	0.03	< dl	0.99	8.71	11.42
Root 4	0.17	0.41	0.60	0.18	0.03	1.37	9.44	14.55
Root 5	0.08	0.28	0.64	0.07	< dl	1.07	7.47	14.34
Root 6	0.06	0.27	0.23	0.08	< dl	0.64	8.00	8.01
Root 7	0.13	0.59	0.86	0.26	0.04	1.89	9.81	19.24
Root 8	0.25	0.83	1.55	0.51	0.03	3.17	3.72	85.17
Root 9	0.2	1.07	2.09	0.53	0.07	3.96	9.67	40.99
Root 10	0.06	0.23	0.34	0.03	< dl	0.66	7.8	8.4
Root 11	0.09	0.48	0.87	0.04	< dl	1.48	2.54	58.39

References

1. *GH/T 1135-2017*; Selenium Rich Agricultural Products. GH-China Supply and Marketing Cooperation General Association: Beijing, China, 25 April 2017.
2. Bañuelos, G.S.; Walse, S.S.; Yang, S.I.; Pickering, I.J.; Fakra, S.C.; Marcus, M.A.; Freeman, J.L. Quantification, Localization, and Speciation of Selenium in Seeds of Canola and Two Mustard Species Compared to Seed-Meals Produced by Hydraulic *Press. Anal. Chem.* **2012**, *84*, 6024–6030. https://doi.org/10.1021/ac300813e.
3. Amoako, P.O.; Kahakachchi, C.L.; Dodova, E.N.; Uden, P.C.; Tyson, J.F. Speciation, Quantification and Stability of Selenomethionine, S-(Methylseleno) Cysteine and Selenomethionine Se-Oxide in Yeast-Based Nutritional Supplements. *J. Anal. At. Spectrom.* **2007**, *22*, 938–946. https://doi.org/10.1039/b703381h.
4. Shi, W.; Hou, Y.; Zhang, Z.; Yin, X.; Zhao, X.; Yuan, L. Determination of Selenium Speciation in High Se-Enriched Edible Fungus Ganoderma Lucidum Via Sequential Extraction. *Horticulturae* **2023**, *9*, 161. https://doi.org/10.3390/horticulturae9020161.
